# Congenital syphilis: associated factors in a follow-up outpatient clinic

**DOI:** 10.1590/1984-0462/2023/41/2022049

**Published:** 2023-05-29

**Authors:** Janer Aparecida Silveira Soares, Ana Paula Ferreira Holzmann, Bárbara Bispo da Silva Alves, Caio Fagundes Quadros Lima, Antônio Prates Caldeira

**Affiliations:** a Universidade Estadual de Montes Claros, Montes Claros, MG, Brazil.

**Keywords:** Syphilis, Congenital syphilis, Sexually transmitted diseases, Follow-up care, Prenatal care, Sífilis, Sífilis congênita, Doenças sexualmente transmissíveis, Seguimento assistencial, Assistência pré-natal

## Abstract

**Objective:**

This study aimed to describe the characteristics of mothers and children assisted in a follow-up clinic for congenital syphilis and identify the factors associated with the confirmation of the diagnosis.

**Methods:**

This is a prospective study conducted from 2016 to 2019 in Montes Claros, Northern Minas Gerais, Brazil. Specific forms addressing maternal sociodemographic, behavioral, and lifestyle habit characteristics, as well as characteristics related to access to healthcare, were used. Hierarchical Poisson regression analysis was performed to define the factors associated with diagnostic confirmation, including the calculation of the prevalence ratios (PR) and respective 95% confidence intervals (95%CI).

**Results:**

A total of 200 binomials (mother-child) who attended at least one appointment as part of the follow-up after discharge from the maternity hospital were eligible for the study. The mothers were mostly young (79.0%), with a low educational level (43.0%), and black (89.5%). Nearly half of the mothers reported not having a steady sexual partner (42.5%). About a quarter attended less than six prenatal appointments (27.5%). Nearly half did not treat the disease adequately during pregnancy (24.5%). The diagnosis of congenital syphilis was confirmed for 116 children. The following factors were associated with the diagnostic confirmation after multiple analyses: low maternal educational level (PR 1.30; 95%CI 1.05–1.60), maternal risky sexual behavior (PR 1.34; 95%CI 1.07–1.66), inadequate treatment of the mother (PR 3.16; 95%CI 2.42–4.47), and lack of treatment of the partner (PR 1.44; 95%CI 1,18–1.81).

**Conclusions:**

Syphilis remains a major challenge. The results highlight the social inequities associated with congenital syphilis and the lack of proper management of pregnant women and their partners.

## Resumo

**Objetivo:**

Descrever as características de mães e crianças atendidas em um ambulatório de acompanhamento de sífilis congênita e identificar os fatores associados à confirmação do diagnóstico.

**Métodos:**

Trata-se de um estudo prospectivo realizado de 2016 a 2019, em Montes Claros, norte de Minas Gerais. Foram utilizados formulários específicos que abordavam características sociodemográficas, comportamentais e de hábitos de vida das mães, bem como características relacionadas ao acesso aos cuidados de saúde. Foi realizada análise de regressão de Poisson hierárquica para s definição dos fatores associados à confirmação diagnóstica, com cálculo das razões de prevalência (RP) e respectivos intervalos de confiança de 95% (IC95%).

**Resultados:**

Duzentos binômios (mãe-filho) que compareceram a pelo menos uma consulta como parte do acompanhamento após a alta da maternidade foram elegíveis para o estudo. As mães eram, em sua maioria, jovens (79,0%), com baixa escolaridade (43,0%) e negras (89,5%). Quase metade das mães relatou não ter parceiro sexual fixo (42,5%). Cerca de um quarto compareceu a menos de seis consultas de pré-natal (27,5%). Quase metade não tratou a doença adequadamente durante a gravidez (24,5%). O diagnóstico de sífilis congênita foi confirmado em 116 crianças. Os seguintes fatores foram associados à confirmação diagnóstica após análise múltipla: baixa escolaridade materna (RP 1,30; IC95% 1,05–1,60), comportamento de risco materno (RP 1,34; IC95% 1,07–1,66), tratamento inadequado da mãe (RP 3,16; IC95% 2,42–4,47) e falta de tratamento do companheiro (RP 1,44; IC95% 1,18–1,81).

**Conclusões:**

A sífilis continua sendo um grande desafio. Os resultados revelam as iniquidades sociais associadas à sífilis congênita e a falta de manejo adequado das gestantes e seus parceiros.

## INTRODUCTION

Syphilis may present unspecific clinical manifestations or imitate other infections, and, when pregnant women are infected, the conceptus can be affected, causing serious health damage and even death.^1^ According to the World Health Organization (WHO), syphilis is the second leading cause of stillbirths globally.^2^ However, it is worth noting that more than 70% of children born infected will be asymptomatic. This aspect highlights the importance of adequate follow-up for infected pregnant women and children as well.^2,3^ Additionally, since 2013, there has been a worldwide increase in the indicators of acquired, gestational, and congenital syphilis.^4^

Most cases of syphilis are found in low- and middle-income countries, and Brazil is in line with the increase in the global indicators, although both congenital syphilis and syphilis in pregnant women require mandatory reporting in Brazil, a strategy adopted as an early intervention to reduce the vertical transmission of the disease.^2–4^ In 2019, the detection rate of syphilis in pregnant women was 20.8/1,000 live births, and the incidence rate of congenital syphilis was 8.2/1,000 live births, reflecting a possible improvement in the reports but at the same time showing that there is still a long way to go until the disease is within acceptable parameters.^3^

In Brazil, all neonates born to inadequately treated or untreated mothers with positive serological tests for syphilis during pregnancy or at childbirth have been considered cases of congenital syphilis since 2017 and thus have been investigated and treated as such to control the disease.^5^ The follow-up of those children is crucial for the treatment and evolution control of mothers and children, and it can be particularly useful in guiding preventive measures.^6^

The recent national studies on gestational and congenital syphilis have reported secondary data, stressing the magnitude of the problem.^7,8^ Few studies have focused on the clinical and evolutionary aspects of the disease and on the epidemiological profile of pregnant women and children. Although syphilis has been around for centuries and has well-known clinical characteristics, the profile of the people affected, the burden of the disease, and the therapeutic response must be constantly reevaluated.^9,10^ This study aimed to characterize the profile of mothers and children assisted at a follow-up outpatient clinic for congenital syphilis and to identify the factors associated with the confirmation of the diagnosis.

## METHOD

This is a prospective study with a 4-year follow-up (2016–2019). The study was carried out in the city of Montes Claros, Northern Minas Gerais, Brazil. With an estimated population of about 413,000 inhabitants, the city is an important regional hub, reaching 86 municipalities with healthcare assistance coverage.

The Reference Center for Infectious Diseases (RCID), located in one of the city's polyclinics, has a specialized assistance service (SAE) and a testing and reception center (TRC) to care for patients with STI/AIDS and receive the children from maternity hospitals who were exposed to these diseases for follow-up after hospital discharge.

The target population of this study included children and mothers with a history of gestational syphilis who were referred by local maternity hospitals. The inclusion criteria for the study were as follows: children born to mothers with positive VDRL in any titration and a positive rapid test or a positive FTA-ABS for syphilis before pregnancy, during pregnancy, or at childbirth and puerperium who had not been previously treated and who attended at least one follow-up appointment after discharge from the maternity hospital.

Maternal treatment with penicillin, started and concluded up to 30 days before delivery and whose titer decreased after 3 months, was considered adequate in doses considering the staging of the disease, as recommended by the Brazilian Ministry of Health.^3,5^ The treatment of the partner was considered appropriate when performed with at least 2,400,000 IU of benzathine penicillin or in a dose like that of the pregnant woman.

Pregnant women who presented false-positive tests or serological scars from previous treatment, or whose pregnancy outcome was abortion or stillbirths, were excluded. Mothers and children who did not return for follow-up within 6 months of their last appointment and were not found through an active search [telephone contact, home visit by the family health strategy (FHS), and home visit by RCID social assistance] were considered losses.

For clinical evaluation of the children, the schedule followed was that proposed by the Ministry of Health for childcare appointments: 30 days after birth; 2, 4, 6, 9, 12, and 18 months; and then every 6 months, at least until 2 years of age. The follow-up of the child for at least 18 months after birth was considered effective and complete, with the performance and negativity of two consecutive VDRL examinations at intervals of at least 30 days, the absence of clinical symptoms or signs of disease recurrence, neuropsychomotor development, and age-appropriate weight and height gain. For the final diagnosis of exposure or of congenital syphilis, the Ministry of Health criteria of 2017 were used (mother with inadequately treated or untreated syphilis, regardless of the results of clinical evaluation or complementary exams of the newborn; present clinical manifestations or cerebrospinal fluid or radiological alterations and a positive VDRL, regardless of the maternal treatment history and VDRL titration; VDRL of the newborn greater than the maternal in at least two dilutions, regardless of the maternal treatment record; and persistence of reactive VDRL after 6 months of age or reactive FTA-ABS after 18 months of age without previous treatment).^5,11^

In addition to the clinical evaluation, all children were also referred for fundoscopy every six months, evaluation of otoacoustic emissions (OAEs) at birth and at 6 months of age, followed by at least one audiometry with OAEs+brainstem auditory evoked potentials (BAEPs) at 12 months and/or FTA-ABS after 18 months of age. For children with neurosyphilis, the cerebrospinal fluid (CSF) study six months after the initial treatment was mandatory, and for those children with some bone alteration, X-ray exams were also performed as requested by the orthopedic clinic.

Data collection was conducted prospectively, directly from medical records, using the forms specially designed to address the variables on maternal sociodemographic characteristics (age, education, and marital status), behavioral and lifestyle habits (smoking, alcohol consumption, use of illicit drugs, number of sexual partners during pregnancy, and risk behavior during pregnancy, including any attitude that implies the possibility of unsafe sex), and factors related to access to healthcare and to healthcare services (pregnancy data, prenatal care, exams, maternal treatment, treatment of the partner, and the management of the child at birth).

**Figure 1:**
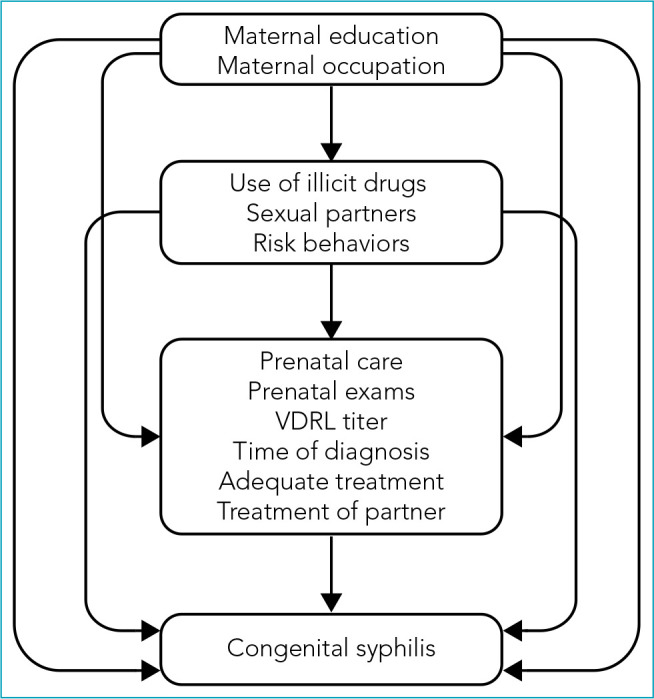
Hierarchical model for analysis of factors associated with congenital syphilis.

The variables were evaluated based on the distribution of absolute and percentage frequencies. The chi-square test or Fisher's exact test was used to define the factors associated with the diagnostic confirmation of congenital syphilis. Multiple analyses were performed on variables that were associated up to the 20% level (p<0.20). A hierarchical Poisson regression analysis was performed to define the factors associated with diagnostic confirmation, defining the prevalence ratios (PR) and respective 95% confidence intervals (95%CI). In the proposed model, distal variables were demographic and social characteristics, intermediate variables were defined by behavioral aspects, and proximal variables were related to health services, care, and treatment (Figure 1). For the final model, only variables with a significance level of 5% (p<0.05) were kept.

All ethical aspects were observed. Mothers involved in the study signed a free and informed consent form. The study was authorized by the Health Department of the Municipality of Montes Claros, and the project was approved by the Research Ethics Committee of the State University of Montes Claros (UNIMONTES) protocol no. 2,341,969.

## RESULTS

During the study period, 234 binomials (mother-child) with a history of syphilis during pregnancy and/or congenital syphilis were sent to RCID for evaluation and follow-up. A total of 34 patients who did not meet the inclusion criteria for the study were excluded. Of them, 11 were excluded for being characterized as a maternal serological scar, three for presenting conflicting tests (rapid test and/or negative FTA-ABS with positive VDRL in repeated tests at intervals of more than 30 days), and 20 for being from other cities. Thus, 200 binomials were eligible for this study.

Table 1 shows the main sociodemographic, behavioral, and healthcare characteristics of mothers of children followed at RCID in the period 2016–2019. The mean age was 24 (SD=5.9) years, ranging from 13 to 44 years. Regarding marital status, 52.5% reported being married or living in a stable relationship with the child's father. As for education, 43.0% of mothers reported having less than 9 years of schooling. The use of illicit drugs during pregnancy (cocaine or crack) was reported by 36 (18.0%) mothers, and having more than one partner was reported by 85 (42.5%) mothers.

**Table 1.** Sociodemographic, behavioral, and healthcare characteristics of mothers of children with suspected congenital syphilis, Montes Claros (MG), 2016–2019.

	n	%
Maternal age (years)		
≤19	50	25.0
20–29	108	54.0
≥30	42	21.0
Marital status		
Married/stable relationship	105	52.5
Single/separated/widowed	95	47.5
Education (years of schooling)		
>12	17	8.5
9–12	97	48.5
<9	86	43.0
Skin color (self-reported)		
White	18	9.0
Brown (mixed race)	148	74.0
Black	33	16.5
Indigenous	1	0.5
Drug use during pregnancy		
Use of any drug (legal/illicit)	53	26.5
Alcohol	32	16.0
Tobacco	28	14.0
Cocaine/crack	36	18.0
None	147	73.5
Partners during pregnancy		
Only one	115	57.5
More than one/no steady partner	85	42.5
Number of prenatal appointments		
> 6	145	72.5
1–6	47	23.5
None	8	4.0
Time of diagnosis		
Before pregnancy	22	11.0
During pregnancy	135	67.5
At childbirth or puerperium	43	21.5
Treatment during prenatal care (UI)		
Benzathine penicillin 7,200,000	111	55.5
Benzathine penicillin 4,800,000	20	10.0
Benzathine penicillin 2,400,000	16	8.0
Ceftriaxone	4	2.0
No treatment	49	24.5
VDRL titers at birth		
1:1–1:8	125	67.6
1:16–1:32	43	23.2
1:64 or over	17	9.2
VDRL testing during prenatal care		
VDRL not tested in the first trimester	30	15.0
VDRL positive in the first trimester	86	50.6
VDRL not repeated during prenatal care	66	33.0
Treatment of the partner		
Yes	85	42.5
No	115	57.5

VDRL: Venereal Disease Research Laboratory.

Regarding prenatal care, eight patients reported not having had at least one appointment during pregnancy. The diagnosis of syphilis was established during prenatal care for 135 (67.5%) mothers. VDRL testing was carried out in the first trimester in 86 (43%) pregnant women and was found negative in 15% (30); 33% of the pregnant women had only one exam during pregnancy and 32.4% had a VDRL titer greater than 1:16 at childbirth.

Table 2 presents the main characteristics of the children followed up: 18 (9.0%) were born under 37 weeks of gestational age, 31 (15.5%) weighed less than 2500g at birth, and 192 (96.0%) were screened for the disease with a non-treponemal peripheral blood test at birth.

**Table 2.** Clinical characteristics, examinations, treatment, and follow-up performed in children exposed to or affected by treponema pallidum at birth.

	n	%
Gestational age at birth (weeks)		
<37	18	9.0
37–41	175	87.5
>41	7	3.5
Apgar in the first minute		
≥7	172	86.0
≤7	28	14.0
Birth weight (g)		
<2500	31	15.5
≥2500	169	84.5
VDRL tested at birth		
Yes	192	96.0
No	8	4.0
Intercurrences at birth		
Absent	176	88.0
Respiratory	10	5.0
Hematological/infectious	5	2.5
Two or more of the above	9	4.5
X-Ray of long bones		
Carried out and no alterations	141	97.2
Periostitis/metaphysitis	3	2.1
Bone fracture	1	0.7
Alterations in the CSF study		
No alterations	136	94.4
VDRL positive	6	4.2
Increase of proteins and/or cells	2	1.4
Treatment of newborn in maternity		
No treatment	12	6.0
Crystalline penicillin 10 days	117	58.5
Procaine penicillin 10 days	19	9.5
Benzathine penicillin single dose	44	22.0
Ceftriaxone EV 10 days	8	4.0
Newborn age at negative VDRL		
Up to 3 months of life	126	63.0
Up to 6 months of life	35	17.5
Did not become negative	1	0.5
Not assessed	38	19.0
Presence of sequelae at 18 months		
No sequelae	95	94.0
Auditory	2	2.0
Cardiological	3	3.0
Cognitive	1	1.0
Outcome according to criteria of Brazilian Ministry of Health (2017)		
Congenital syphilis	116	58.0
Absence of disease	66	33.0
Cannot be established	18	9.0

VDRL: Venereal Disease Research Laboratory; CSF: cerebrospinal fluid; RX: X-ray.

Intercurrences at birth were recorded for 24 (12.0%) children, with respiratory disorders being the most frequent, followed by early neonatal sepsis. An X-ray of the long bones was performed in 145 (72.5%) children, showing some alteration in four newborns. Spinal taps were performed in 144 (72%) children, with 8 of them showing abnormalities.

As for the treatment carried out in the maternity hospital, 90.0% of the children were treated with penicillin and 4.0% with ceftriaxone. For 45 children, management at birth did not follow the Brazilian Ministry of Health recommendations.

The VDRL became negative in the first 30 days of follow-up for 116 children (58.0%). Only one child maintained a positive VDRL at 12 months of age and needed to be retreated; 101 children (50.5%) completed the follow-up recommended by the Ministry of Health, two of whom had a positive FTA-ABS test at 18 months of age.

Considering the 2017 criteria of the Brazilian Ministry of Health, 116 children had a confirmed diagnosis of congenital syphilis. For 66 children, the outcome was only exposure to the disease, and 18 children were lost to follow-up. Among the children who were followed up for at least 18 months, 6 (5.9%) had some sequelae.

Table 3 shows the results of the bivariate analyses between the characteristics of the mothers and the outcome of the diagnosis of congenital syphilis, and Table 4 presents the final model of the multiple analysis, highlighting the factors associated with the diagnostic confirmation of congenital syphilis for the group of children followed.

**Table 3.** Bivariate analysis of sociodemographic, behavioral, and prenatal management characteristics associated with the congenital syphilis outcome according to the criteria of the Ministry of Health.

	Congenital syphilis		p-value
	No	Yes	
Maternal age (years)			0.285
30 or older	07 (26.9%)	19 (73.1%)	
Up to 30	59 (37.8%)	97 (62.2%)	
Maternal education			0.027
Up to 9 years of schooling	14 (24.6%)	43 (75.4%)	
Over 9 years of schooling	52 (41.6%)	73 (58.4%)	
Maternal occupation			0.018
Housewife	26 (28.9%)	64 (71.1%)	
Student/work out of home	41 (44.6%)	51 (55.4%)	
Marital status			0.555
Single/no steady partner	30 (34.2%)	58 (65.9%)	
Married/stable relationship	36 (38.3%)	58 (61.7%)	
Use of illicit drugs during pregnancy			0.007
No	63 (40.1%)	94 (59.9%)	
Yes	03 (12.0%)	22 (88.0%)	
Maternal risky sexual behavior			0.002
No	50(45.0%0	61 (55.0%)	
Yes	16 (32.5%)	55 (77.5%)	
Maternal partners during pregnancy			0.017
Only 1	45 (43.7%)	58 (56.3%)	
More than 1 or no steady partner	21 (26.6%)	58 (73.4%)	
Number of appointments during pregnancy			<0.001
>6	59 (43.7%)	76 (56.3%)	
≤6	07 (14.9%)	40 (85.1%)	
Time of maternal diagnosis			<0.001
Before pregnancy	11 (61.1%)	07 (38.9%)	
During pregnancy	53 (43.8%)	68 (56.2%)	
At childbirth or puerperium	02 (4.7%)	41 (95.3%)	
Repeated VDRL testing during prenatal care			<0.001
2 or more	49 (53.3%)	43 (46.7%)	
Fewer than 2	17 (18.9%)	73 (81.1%)	
Adequate maternal treatment			<0.001
Yes	63 (72.4%)	24 (27.6%)	
No	03 (3.2%)	92 (96.8%)	
Maternal VDRL titer at childbirth			0.035
1:1–1:8	44 (39.6%)	67 (60.4%)	
≥1:16	13 (23.2%)	43 (76.8%)	
Treatment of the partner			<0.001
Yes	44 (58.7%)	31 (41.3%)	
No	22 (20.6%)	85 (79.4%)	

*According to the guidelines of the Ministry of Health. VDRL: Venereal Disease Research Laboratory.

**Table 4.** Factors associated with diagnostic confirmation of congenital syphilis, Montes Claros (MG), 2016–2019 (Poisson regression analysis) (n=182).

	RP (95%CI)	p-value
Maternal education		0.016
≤9 years of schooling	1.30 (1.05–1.60)	
>9 years of schooling	1.0	
Maternal risky sexual behavior		0.008
Yes	1.34 (1.07–1.66)	
No	1.0	
Adequate maternal treatment		<0.001
No	3.16 (2.42–4.47)	
Yes	1.0	
Treatment of the partner		<0.001
No	1.47 (1.18–1.81)	
Yes	1.0	

PR: prevalence ratio; 95%CI: 95% confidence interval.

## DISCUSSION

The group of mothers evaluated was characterized by being predominantly young and having a high proportion of single mothers, or mothers without steady partners. In addition, several mothers had a low education level and were predominantly black, highlighting the association of maternal syphilis with social inequalities.^7,8,11^

The demographic data investigated show that a quarter of the mothers were adolescents, which has implications concerning the maturity of the pregnant woman to care for both her own healthcare and the child's healthcare.^12^ Pregnant women aged between 20 and 39 years accounted for more than 60% of patients affected, which is in line with data from the Ministry of Health by age group showing higher incidences of the disease.^3^

Regarding marital status, being in a stable relationship or married was not a maternal protective factor for acquiring the disease, since over half the mothers fit this status and remained in a condition of vulnerability to the disease, which is in line with other studies.^8,13^

The partner's health condition has no longer been a criterion for reporting congenital syphilis since the 2017 recommendations of the Ministry of Health, but the treatment of the partner is crucial for the control of maternal disease, which places him in such an important role as the others in the congenital syphilis prevention cycle.^5^ In this study, an association was found between the lack of treatment of the partner with a higher occurrence of children with congenital syphilis.

A slightly higher maternal education level was seen in this study compared to others that evaluated the profile of pregnant women with syphilis.^14,15^ However, the fact that the patients had an average of 11 years of schooling had no positive impact on the disease's prevention. It is realized that there is a gap between access to information and the practice of habits and customs that benefit the individual.^16^

The use of illicit drugs was associated with the outcome presence of a diagnosis of congenital syphilis, quite possibly related to behavioral issues in these specific groups of patients, such as having multiple partners, nonadherence to safe sex practices, and a lack of prenatal care. The indices found here are above the national average.^15^

Unlike other studies that have indicated HIV as a comorbidity often associated with syphilis, in this study, none of the mothers had this association. This unusual finding surprised the authors. It is possible that the sample size was not large enough to represent this population, since the prevalence of pregnant women with this infection in the evaluated service is unknown.

As in other similar studies,^7,8^ it was observed that most pregnant women reported prenatal care appointments. However, the appointments are not a guarantee of quality prenatal care and adequate management. Previous studies show that, although there has been an increase in the number of appointments and that prenatal care has started earlier, as well as the performance of rapid tests, the scenario of the disease in the country has not improved.^7,8,15^ There is an urgent need to provide continuing education activities for health professionals, especially in the context of primary care, to avoid gaps and missed opportunities for effective interventions to control syphilis.^17^

In this study, about one-third of the pregnant women were treated at childbirth or puerperium, whether due to late diagnosis, failures in prenatal diagnosis, or difficulty to treat. Although 100% of the municipality's basic health units have provided rapid tests for syphilis since the end of 2018 using the reverse approach algorithm, only two health units have delivered the specific medication on an outpatient basis, and this may be a factor that hinders the treatment.

The absence of adequate maternal treatment was associated with the outcome of congenital syphilis. Other studies have shown that the time the treatment starts during pregnancy can reduce perinatal mortality due to the disease by up to 70%, when it is started before 21 weeks of gestation.^12,18^ These data further reinforced the importance of diagnosis, access to treatment, and knowledge of health professionals in managing the disease in order to take effective action.^19^ It is worth highlighting the need for training of the neonatal care team because some children were not tested at birth and others did not receive adequate treatment.

In this follow-up, it was found that the referred children did not present early manifestations suggestive of the disease, which were not included in the hospital discharge reports either, such as serosanguineous coryza, hepatomegaly, splenomegaly, hemolytic anemia, cholestasis, petechiae, and/or rash. These data may represent a limitation of this study, considering that mild conditions can have subtle manifestations that may be missed by health professionals since most children were treated at birth, and some studies address the possibility of lower vertical transmission of the disease in children born to mothers treated early in pregnancy.^10^

Similarly, radiological findings typical of congenital syphilis were also uncommon. These findings can be attributed to the fact that most pregnant women used at least one dose of penicillin during pregnancy, even when they were not adequately treated.^20–22^

Of the children who were followed up, only two had a positive treponemal test. According to literature reports, early treatment of the conceptus induces faster negative serological test results, and the absence of a positive test does not exclude the disease. However, a positive test after 18 months confirms a diagnosis.^11,20,21^ It is noteworthy here that 6 of the 101 children followed had some sequelae possibly associated with syphilis, and, in these cases, the follow-up played a relevant role in early interventions.

In this study, as in other similar ones, it is observed that the newborn is often treated “just in case”, once the recommended treatment does not present any relevant adverse effects.^20,23^ Although treatment often exceeds the recommendation of the Ministry of Health, it is a common practice because some studies point to the loss of follow-up as frequent and early.^20,23^

In our study, the greatest loss of children to follow-up occurred up to the sixth month of age, most likely after two negative VDRL test results. Perhaps the caregivers might have interpreted that, in the absence of positive tests, the follow-up would no longer be necessary and the child would be free from the disease. As seen in other studies, the loss to follow-up is higher than expected, although the follow-up of such children is crucial for the diagnostic conclusion and immediate preventive actions to minimize adverse outcomes.^9,22^

It is important to consider some limitations of this study, in addition to the lack of follow-up already mentioned. It is not possible to ensure that all referred mothers effectively seek the service. Additionally, the factors identified are restricted to the group being monitored and cannot be generalized. Even so, the study has the merit of pointing out the magnitude of the problem and critical situations that must be promptly evaluated.

Characterizing the information on diseases related to syphilis, whether it is gestational or congenital, is important to guide practices concerning healthcare, maternal-infant health, and epidemiological surveillance, contributing to foster actions aiming to reduce the disease and keep it under control.^12^ These are especially relevant as congenital syphilis is considered a sentinel event in the quality of prenatal care.^7,8,12^

The characteristics of the evaluated group point to precarious socioeconomic conditions and access to goods and services. Factors associated with the diagnosis of congenital syphilis were low maternal education level, maternal risky sexual behavior, inadequate maternal treatment, and lack of treatment of the partner. These results highlight the social inequities associated with congenital syphilis and the lack of proper management of pregnant women and treatment of partners.

## Data Availability

The database that originated the article is available with the corresponding author.
